# Copper-Containing Bionanocomposites Based on Natural Raw Arabinogalactan as Effective Vegetation Stimulators and Agents against Phytopathogens

**DOI:** 10.3390/polym16050716

**Published:** 2024-03-06

**Authors:** Spartak S. Khutsishvili, Alla I. Perfileva, Tatyana V. Kon’kova, Natalya A. Lobanova, Evgeniy K. Sadykov, Boris G. Sukhov

**Affiliations:** 1 Rafael Agladze Institute of Inorganic Chemistry and Electrochemistry, Ivane Javakhishvili Tbilisi State University, 11 Mindeli St., 0186 Tbilisi, Georgia; 2 Laboratory of Plant-Microbe Interactions, Siberian Institute of Plant Physiology and Biochemistry, Siberian Branch of the Russian Academy of Sciences, 664033 Irkutsk, Russia; alla.light@mail.ru; 3 Laboratory of Nanoparticles, V. V. Voevodsky Institute of Chemical Kinetics and Combustion, Siberian Branch of the Russian Academy of Sciences, 630090 Novosibirsk, Russia; konbuivol_2@yahoo.com (T.V.K.); boris_sukhov@mail.ru (B.G.S.); 4 Laboratory of Unsaturated Heteroatomic Compounds, A. E. Favorky Irkutsk Institute of Chemistry, Siberian Branch of the Russian Academy of Sciences, 664033 Irkutsk, Russia; lona@irioch.irk.ru; 5 Laboratory of Metal-Organic Coordination Polymers, A. V. Nikolaev Institute of Inorganic Chemistry, Siberian Branch of the Russian Academy of Sciences, 630090 Novosibirsk, Russia; sadykov@niic.nsc.ru

**Keywords:** nanocomposite, arabinogalactan, copper, in vitro, agricultural plants, *Clavibacter sepedonicus*

## Abstract

Novel copper-containing bionanocomposites based on the natural raw arabinogalactan have been obtained as universal effective agents against phytopathogen *Clavibacter sepedonicus* and development stimulants of agricultural plants. Thus, the use of such nanosystems offers a solution to the tasks set in biotechnology while maintaining high environmental standards using non-toxic, biocompatible, and biodegradable natural biopolymers. The physicochemical characteristics of nanocomposites were determined using a number of analytical methods (elemental analysis, transmission electron microscopy and spectroscopic parameters of electron paramagnetic resonance, UV–visible, etc.). The results of the study under the influence of the nanocomposites on the germination of soybean seeds (*Glycine max* L.) and the vegetation of potatoes (*Solanum tuberosum* L.) showed the best results in terms of biometric indicators. It is especially worth noting the pronounced influence of the nanocomposite on the development of the root system, and the increase in the mass of the potato root system reached 19%. It is also worth noting that the nanocomposites showed a stimulating effect on the antioxidant system and did not have a negative effect on the content of pigments in potato tissues. Moreover, the resulting bionanocomposite showed a pronounced antibacterial effect against the phytopathogenic bacterium. During the co-incubation of phytopathogen *Clavibacter sepedonicus* in the presence of the nanocomposite, the number of cells in the bacterial suspension decreased by up to 40% compared to that in the control, and a 10% decrease in the dehydrogenase activity of cells was also detected.

## 1. Introduction

Today, agriculture is facing serious problems associated with the need for optimal plant nutrition during the growing season. Considering the growing demand of the world market, great efforts in biotechnology are aimed at developing a new generation of complex organomineral fertilizers [1,2,3,4]. Such fertilizers should improve the assimilation of microelements by plants in safe doses, ensure their prolonged action, and be resistant to leaching of microelements from various types of soils. In addition, the search for more efficient and safe fertilizers is becoming an increasingly urgent task, especially in the context of climate change and the relevance of healthy nutrition. Achievements in the development of new materials and methods of using nanotechnologies to improve the efficiency of agricultural production are widely covered in scientific publications [5,6,7]. The use of nanomaterials as microfertilizers and plant protection products helps to increase plant resistance to adverse weather conditions, reduce morbidity, and increase stress resistance. Such organomineral nanosystems make it possible to obtain a larger yield from the same areas, there is a better absorption of nutrients by plants, and the use of fertilizers in the form of nanosubstances is carried out in smaller quantities compared to the salts used and also minimizes their loss to the environment.

Biopolymer nanomaterials seem to be one of the successful solutions for these problems, and first of all, it is worth noting nanobiocomposites (NCs) based on polysaccharides. Such biopolymers act as effective stabilizing matrices of nanosized metal particles capable of forming new functional materials, including for the design of fertilizers based on metal-containing NCs [8,9,10]. At the same time, nanochemistry also faces challenges such as the creation of biocompatible, safe, and biologically easily degradable nanosubstances, so the use of natural polysaccharides as matrices is more than relevant [11,12,13]. The use of water-soluble natural raw polysaccharide arabinogalactan (AGr), enriched with flavonoids, is promising both for the successful solution of synthetic problems in the formation of multifunctional NCs and for applications in biotechnology and agriculture. The matrix of natural polysaccharide arabinogalactan possesses valuable properties such as biocompatibility, non-toxicity, hydrogel-forming ability [14], enantio-selective tautomeric hydrotropism for hydrophobic antiradical flavonoids [15,16], etc. [17].

In turn, copper (Cu) plays an important role in the biochemical processes of plants. A deficiency or excess of Cu microelements can cause serious diseases and low yields [18]. For example, Cu is involved in photosynthesis, is responsible for plant resistance to bacterial and fungal diseases, is contained in copper-containing enzymes, and determines their activity [19]. Cu also plays a key role in various enzymatic reactions, photosynthetic and respiratory chains (in electron transport), cell membrane function, and protective processes against oxidative stress [20,21]. Modern NCs, including metal-containing particles, have increased stability to external influences, change solubility, possess higher biological activity, etc., and the synergy of the properties of the polymer matrix and metal-containing nanoparticles provides new opportunities [8,22]. Thus, copper-containing NCs based on polysaccharides are used for a pre-sowing seed treatment [23,24]. The microelement increases germination, increases productivity, reduces the diseases of seeds, and, at the same time, has a sparing effect on the biocenosis of fields [25]. Also, copper-containing NCs are used as growth stimulants for plants and as agents that increase plant immunity [26].

Nanomaterials used in various methods of healing and cultivating plants are a promising direction and are intended to stimulate plant growth in open or protected ground conditions. At the same time, modern biotechnology is inextricably linked with the cultivation of plants on artificial nutrient media that have a balanced composition of nutritional components necessary for the growth and development of plants in large quantities, especially plants that are difficult to grow under normal conditions. The use of artificial nutrient media is of great importance in the design of autonomous life support systems, for example, under conditions of long-term space flights [27,28]. All this involves the use of high-tech methodology, developing plant cultivation techniques based on safety and biodegradable materials in order to obtain planting material free from various phytopathogens. This means that an important goal is to obtain multifunctional NCs that inhibit biofilm formation and exhibit an antimicrobial effect against phytopathogens. Thus, the bacterium *Clavibacter sepedonicus* (*Cms*) is a quarantine object in most countries of the world and causes serious crop losses (up to half) [29,30]. The infection is latent in nature and manifests itself in the form of the wilt and yellowing of stems during the growing season. The problem is exacerbated by the lack of effective methods against this bacterium; all measures are only preventive and are associated with the processing of equipment and the manual removal of diseased plants. There are also physicochemical methods against *Cms*, for example, using temperature treatment [31], which is difficult to implement on a large scale and while maintaining the viability of plants. Therefore, the search for an agent to regulate the number of this pathogen is extremely important. Previously, we demonstrated the success of using a number of nanocomposites based on natural polysaccharides with nanoparticles of chalcogens and metals [4,12,32,33]. Thus, promising results were obtained on the effect of similar composites with selenium-, manganese-, silver-, and sulfur-containing nanoparticles on potato vegetation and productivity both in field conditions as well as on biometric parameters of plants and resistance to biotic stress in laboratory experimental conditions, including biological activity composites on the inhabitants of the rhizosphere.

The manuscript presents the results of the obtained copper-containing nanobiocomposite (AGr-Cu) based on AGr, which has shown itself to be a multifunctional and promising agent capable of solving many of the problems in modern agriculture. Thus, the synthesized NC is able to function as effective and safe microfertilizers, an antimicrobial agent against phytopathogenic *Cms*, as well as to stimulate the plant growth and seed germination of cultivated plants. Using the model system of potatoes (*Solanum tuberosum* L.) in vitro, we have studied the qualitative and quantitative influence of the copper-containing NC on plant growth and obtained additional information on the development of the plants. Potatoes were chosen as the object of research, since they are a sensitive plant to Cu content [34,35]. The in-depth study of metabolism occurring in potatoes (one of the most important agricultural crops, *Solanum tuberosum* L.) and their optimal nutrition are of great interest to biochemists and plant physiologists. In addition, as discussed above, such copper-containing NCs may be promising for seed treatment, so it was also decided to test the resulting AGr-Cu as growth stimulants in vitro on *Glycine max* L. soybean seeds.

## 2. Results

### 2.1. Structural Features and Physicochemical Properties of Obtained AGr-Cu

To synthesize AGr-Cu, the method of chemical reduction of CuCl_2_ with sodium borohydride in an aqueous solution of the polysaccharide was used using the conformational voids of AGr macromolecules as nanoreactors for the synthesis of nanosized particles. The organomineral composites obtained as a result of the redox reaction are water-soluble compounds in which spherical copper (I) oxide nanoparticles are formed and stabilized by a polysaccharide matrix. The most probable composition of the nanoparticles formed in the polysaccharide matrix is in the form of hydrated particles of copper oxide Cu_2_O × nH_2_O, which are stabilized in the polymer mass on oxygen atoms and hydroxyl groups by analogy with other polysaccharides [36,37].

**Figure 1:**
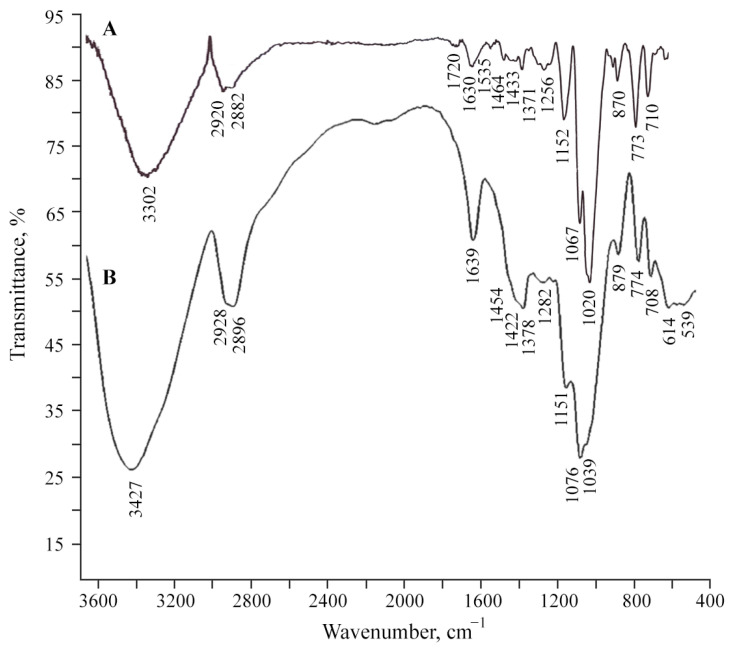
FTIR spectra of (**A**) original polysaccharide AGr and (**B**) composite AGr-Cu.

The studied polysaccharide AGr has a high tendency to form corresponding coordination biopolymer complexes with ions of monovalent and divalent metals, which are located on the surface of metal-containing nanoparticles. According to infrared (FTIR) spectroscopy, the formation of the studied NC is not accompanied by structural changes in AGr (Figure 1). However, a comparative analysis of the FTIR spectra of samples of AGr and AGr-Cu showed that in the high-frequency region, there is a broadening of the absorption region 3000–3600 cm^−1^, caused by stretching vibrations of hydroxyl groups *δ*(OH), indicating a complex formation with Cu^+2^ and the presence of bound water [38,39,40]. The broadening of OH stretching vibrations during complexation is associated with the participation of sugar OH groups in metal–ligand bonding. It should be noted that the main broadenings of OH stretching vibrations in the spectra of the NC are due to the presence of a strong hydrogen network between the OH groups of the AGr polysaccharide matrix and H_2_O molecules in the metal-polysaccharide crystal structures, which are responsible for the stabilization of solid structures and for the retention of aggregated units. Such a system of hydrogen bonds was found in the crystal structure of the Mn(D-gluconate)_2_ × 2H_2_O salt and previously obtained manganese-containing NCs [4,41], which is also associated with ionization due to complexation. In addition, the maximum of the absorption band from *δ*(OH) vibrations (at 1433 cm^−1^), characteristic of one of the more possible positions of the −CH_2_OH group, is shifting towards lower frequencies, which also evidences ionization owing to complexation [40].

**Figure 2:**
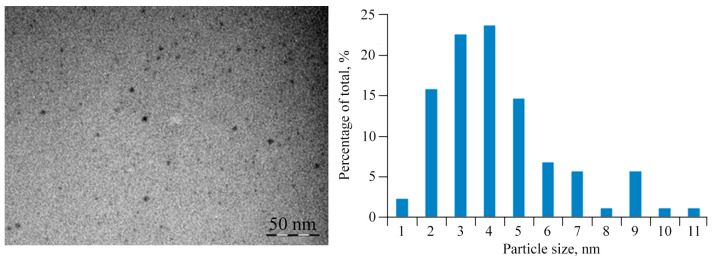
Typical TEM micrograph and size distribution of nanoparticles of AGr-Cu. The scale size is 50 nm.

**Figure 3:**
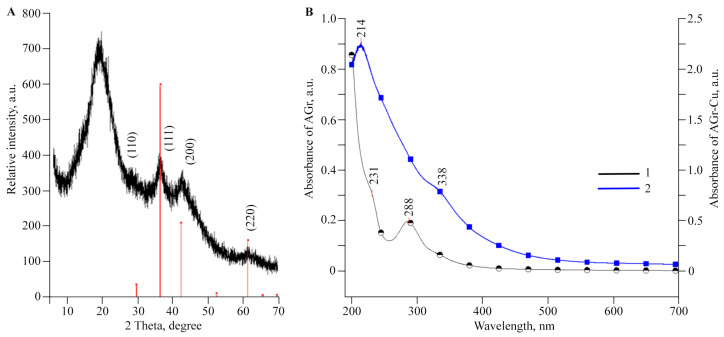
The (**A**) XRD pattern of AGr-Cu and (**B**) UV–visible spectra of (1) AGr and (2) AGr-Cu.

According to transmission electron microscopy (TEM), the sizes of copper (I) oxide nanoparticles in AGr-Cu are in the range of 1–26 nm, with predominant particles with sizes of 2–5 nm (Figure 2). X-ray phase analysis (XRD) data for AGr-Cu show in the range of 10–25 θ,a halo from the amorphous polysaccharide matrix, and a phase of Cu_2_O crystallites [42], with average particle sizes also in the range of 3–4 nm (Figure 3A), determined by the Scherrer formula [43]. Note that the diffraction peaks (110), (111), (200), and (220) characteristic of Cu_2_O are observed in the X-ray diffraction patterns, while no other peaks (such as CuO or Cu) were detected.

The UV–visible spectrum of AGr-Cu (Figure 3B) contains an absorption band in the region of 338 nm, which is caused by the surface plasmon resonance of Cu_2_O nanoparticles. According to the literature data, this absorption peak for Cu_2_O nanoparticles is observed in a wide range of wavelengths (300–500 nm) [44,45], which is explained by the geometric characteristics of the particles and their different morphologies. The optical absorption of AGr in the region of 225–330 nm can be attributed to the flavonoids present in AGr, the predominant of which is taxifolin (dihydroquercetin) [46], which in turn also has three peaks in the optical absorption curve at 230, 290, and 330 nm [47], almost coinciding with the observed three peaks in this region for AGr (Figure 3B).

**Figure 4:**
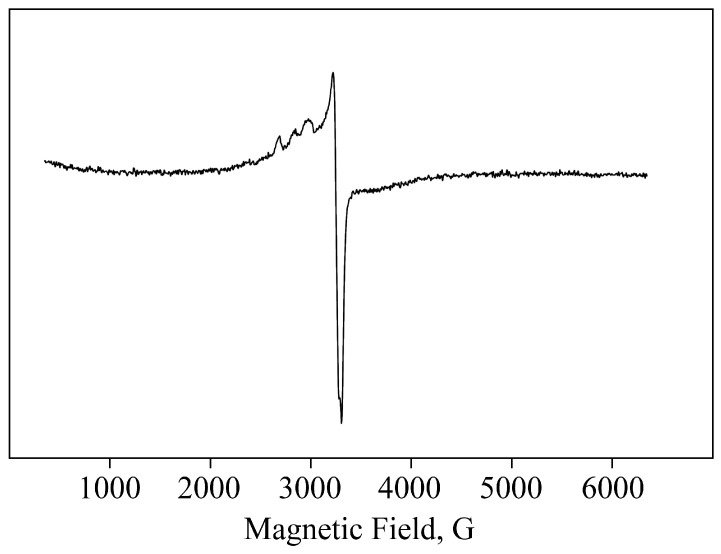
The EPR spectrum of AGr-Cu.

Monovalent Cu has a 3*d*^10^ electron configuration with no unpaired electrons, making it undetectable by electron paramagnetic resonance (EPR). However, the EPR spectra for AGr-Cu contain signals characteristic of Cu^+2^ complexes. The 3*d*^9^ configuration of Cu^+2^ ions means that its compounds are paramagnetic, making EPR a useful tool for structural compounds containing Cu(II). Due to the relatively broad individual line (Δ*H* ≈ 80 G), only the hyperfine structure (HFS) of Cu^+2^ nuclei (*I* = 3/2) in parallel orientation (with *g*_ǁ_ = 2.63–2.70 and *A*_Cu_ = 125–176 Gs) is clearly resolved in the spectra [48] (see Figure 4). In a perpendicular orientation, the spectral lines give an unresolved line with an effective *g*-factor in the region *g*_┴_ ≈ 2.15–2.20. These values are within the range typical for Cu(II) complexes in almost square planar coordination [49,50].

### 2.2. The Effect of AGr-Cu on the Growth and Development of Solanum tuberosum L.

**Figure 5:**
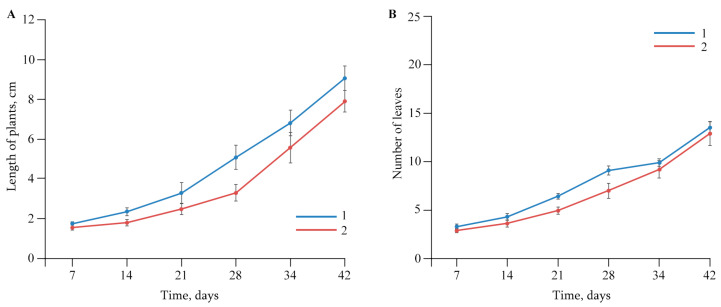
Dynamics of the biometric parameters of (**A**) length of plants and (**B**) number of leaves in (1) control and (2) plants grown in a nutrient medium with AGr-Cu.

Experiments showed that AGr-Cu did not have a negative effect on potato plants *Solanum tuberosum* L.in vitro: on plant growth, number of leaves (Figure 5), biomass of the vegetation part of plants and roots (Table 1). It is worth noting that AGr-Cu also did not have a negative effect on the length of potato internodes in vitro, so in the control, the length was 0.89 ± 0.03 cm, and in the case of AGr-Cu, 0.84 ± 0.03 cm. At the same time, in the presence of NCs, the biomass of potato roots slightly increased.

In order to evaluate the effect of AGr-Cu on potatoes, we previously studied the toxic effect on the development of potato plants or with those Cu deficiency in the nutrient medium [51]. Thus, it was shown that there is no excess accumulation of Cu or mineral starvation in plant tissues, and its amount is comparable to that in the control (Table 2).

An important indicator of the viability of a green plant is the functioning of its photosynthetic apparatus, the main component of which is pigments. We investigated the content of pigments in the tissues of potato plants, both those infected with *Cms* and those free from infection in combination with NC treatment (Table 3). It was found that infection of plants with the pathogen led to a significant decrease in chlorophyll *a* and *b* concentrations compared to those in the control. Exposure of uninfected plants to AGr-Cu did not lead to a pronounced effect on the content of photosynthetic pigments. At the same time, the concentration of photosynthetic pigments of infected potato plants treated with the NC was significantly better compared to similar values in infected plants without treatment with AGr-Cu (Table 3).

### 2.3. Evaluation of Potato Root Viability In Vitro in the Presence of AGr-Cu

**Figure 6:**
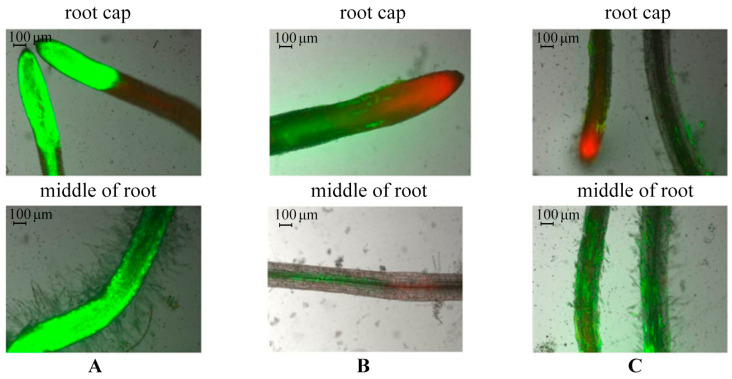
Double-staining of potato root tissues in vitro with fluorescent dyes: (**A**) control, (**B**) plants infected by *Cms*, and (**C**) plants incubated in presence of AGr-Cu. Dead cells are colored red; living cells are green.

The viability of potato roots was evaluated in vitro using double-staining with fluorescent dyes fluorescein diacetate (FDA) and propidium iodide (PI). To do this, we compared samples of control plants, plants infected by *Cms* bacterium, and plants incubated with AGr-Cu for 5 days (Figure 6).

According to the results of microscopy, it was revealed that in the control plants, normal root development was observed: active development of the root cap, root hair zone, and intensive cell division, as evidenced by the green color of the tissues (see Figure 6A). In the infected plants, in the root cap zone, cell death was observed, as evidenced by a red glow. There was also about 60–70% loss of root cell viability and a partial absence of root hairs compared to the control (Figure 6B). When plants were incubated with AGr-Cu, root hairs seemed less developed compared to those in the control, but their viability remained. The presence of dead cells was observed in the root cap, but their number was lower than that during infection, which may be due to physiological processes occurring during root growth and development (Figure 6C).

### 2.4. The Effect of AGr-Cu on the Stress Resistance of Solanum tuberosum L.

**Figure 7:**
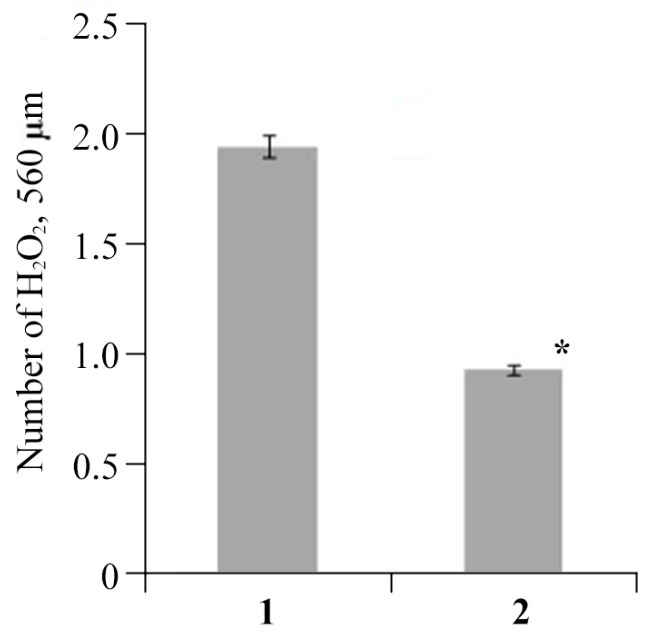
The number of ROS in root tissues in (**1**) control and (**2**) treated with AGr-Cu. The (*) symbol in the graph marks significant differences according to the Mann–Whitney test (*p* ≤ 0.01).

At the first stage of research, to evaluate the effect of the NC on the status of the antioxidant system (AOS) of plants, the accumulation of reactive oxygen species (ROS) in potato root tissues was studied. In the variant with AGr-Cu, the effect of reducing the amount of ROS compared to that in the control was clearly demonstrated (Figure 7). The content of ROS, namely, hydrogen peroxide, in plant samples was determined using xylenol orange dye [52]. The method for determining hydrogen peroxide is based on the oxidation of iron ions Fe^+2^ with hydrogen peroxide to iron ions Fe^+3^, which form colored compounds with xylenol orange. The hydrogen peroxide content was determined spectrophotometrically.

**Figure 8:**
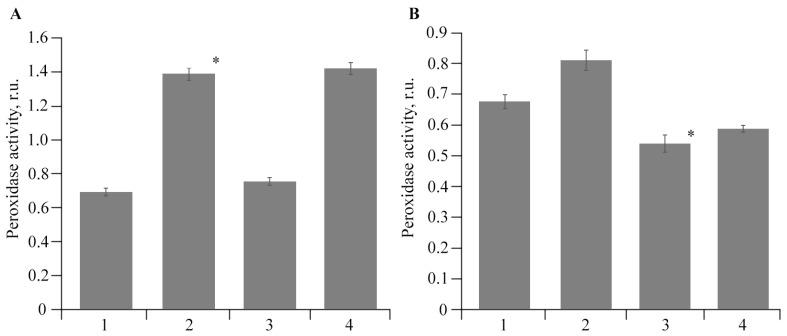
Effect of *Cms* and AGr-Cu on the peroxidase activity in (**A**) potato roots and (**B**) leaves in vitro; 1—control, 2—plants infected by *Cms*, 3—AGr-Cu without *Cms*, 4—infected plants in presence of AGr-Cu. The (*) symbol in the graphs marks significant differences according to the Mann–Whitney test (*p* ≤ 0.01).

To neutralize ROS, AOS enzymes are required, and such enzymes include peroxidase and catalase. It was found that the infection of plants with the pathogen led to a significant increase in peroxidase activity in both the root and leaf tissues of potatoes (Figure 8). Moreover, this effect was more pronounced in root tissues. The peroxidase activity in the tissues of roots and leaves of uninfected plants grown in a medium with AGr-Cu was at the control level. At the same time, in plants infected by *Cms* in the presence of the NC, the peroxidase activity in leaves was lower than that in infected potatoes (Figure 8).

**Figure 9:**
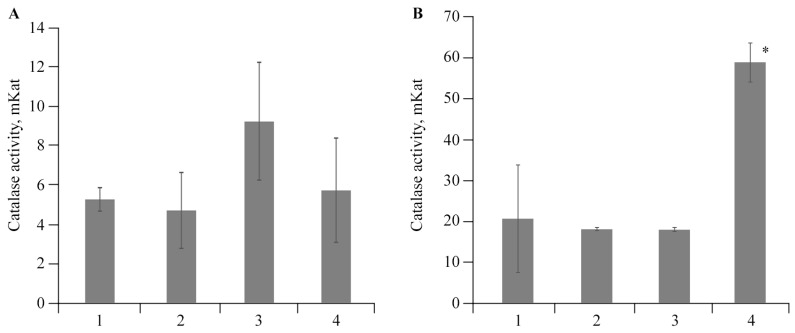
Effect of *Cms* and AGr-Cu on the catalase activity in (**A**) potato roots and (**B**) leaves in comparison with the control in vitro, as well as in plants infected by *Cms* pathogen; 1—control, 2—plants infected by *Cms*, 3—AGr-Cu without *Cms*, 4—infected plants in presence of AGr-Cu. The (*) symbol in the graphs marks significant differences according to the Mann–Whitney test (*p* ≤ 0.01).

To evaluate the effect of stress, we studied the catalase activity in the tissues of roots and leaves in vitro (see Figure 9). After 2 days of NC treatments on infected plants, a pronounced effect of AGr-Cu on catalase activity was shown. This NC significantly increased the enzyme activity in both the leaves and roots of potato plants.

**Figure 10:**
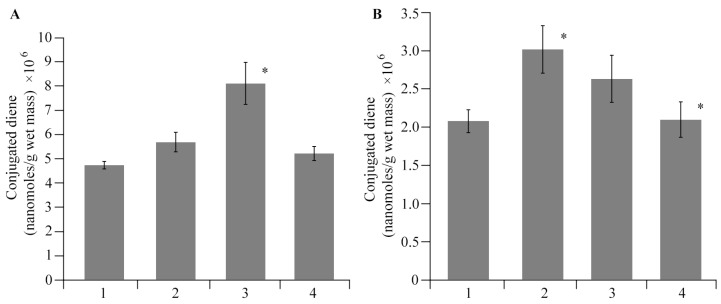
Effect of AGr-Cu treatments on the content of DCs in potato (**A**) roots and (**B**) leaves in comparison with the control in vitro, as well as in plants infected by *Cms* pathogen; 1—control, 2—plants infected by *Cms*, 3—AGr-Cu without *Cms*, 4—infected plants in presence of AGr-Cu. The (*) symbol in the graphs marks significant differences according to the Mann–Whitney test (*p* ≤ 0.01).

A high amount of ROS can lead to negative and irreversible processes occurring in the plant cell. Under any stress, the plant cells produce ROS, messenger molecules that trigger a cascade of protective programs in a cell and are capable of destroying the cell itself and its structures, thus causing lipid peroxidation (LPO), the primary product of which is diene conjugates (DCs). The observed picture is quite understandable, since it is known that the ROS level rises significantly at the initial stage of the plant’s response to the stress factor [53,54]. In this regard, to evaluate the stress level on plants under the AGr-Cu treatments and phytopathogenic infection, we measured the DC content in the tissues of roots and leaves. The results obtained show (Figure 10) that in plants infected by *Cms*, the DC content in roots and leaves tissues increased compared to that in the control. When treated with AGr-Cu, an increase in DC concentration was observed in the root tissues of healthy potatoes; no changes were found in the leaf tissues. After treating infected plants with AGr-Cu, the DC concentration did not change compared to the control in both roots and leaves.

**Figure 11:**
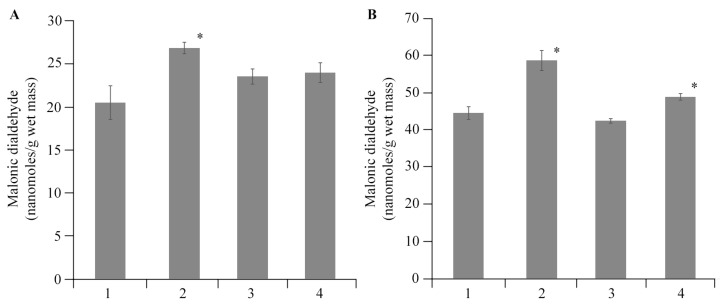
Effect of AGr-Cu treatments on the content of MDA in potato (**A**) roots and (**B**) leaves in comparison with the control in vitro, as well as in plants infected by *Cms* pathogen; 1—control, 2—plants infected by *Cms*, 3—AGr-Cu without *Cms*, 4—infected plants in presence of AGr-Cu. The (*) symbol in the graphs marks significant differences according to the Mann–Whitney test (*p* ≤ 0.01).

Plants respond to infection by increasing the production of ROS, which are subsequently dismutated to hydrogen peroxide by superoxide dismutase. LPO products, along with other substances, include highly reactive dialdehydes such as malonic dialdehyde (MDA), which is found in low concentrations. AGr-Cu did not affect the MDA content in potato tissues (Figure 11).

### 2.5. Antibacterial Activity of AGr-Cu against Cms Phytopathogen

**Figure 12:**
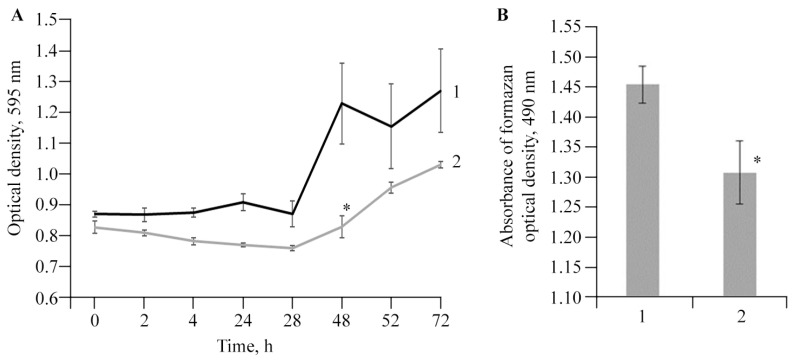
Effect of AGr-Cu treatments on (**A**) growth and (**B**) dehydrogenase activity of *Cms* pathogen compared to control; 1—control, 2—*Cms* in presence of AGr-Cu. The (*) symbol in the graphs marks significant differences according to the Mann–Whitney test (*p* ≤ 0.01).

To evaluate the bacteriostatic effect of antibacterial agents and the viability of bacteria, including anaerobic ones, an indicator such as dehydrogenase activity is widely used [55,56]. Dehydrogenases are directly involved in bacterial respiration, performing various transport functions in the respiratory chain. Therefore, experiments were performed to determine the bactericidal effectiveness of the resulting NC as a potential antibacterial agent against *Cms*. The results showed that AGr-Cu slightly reduced the viability of *Cms* (Figure 12A). At the same time, AGr-Cu significantly reduced the dehydrogenase activity (Figure 12B), which indirectly indicates a decrease in the intensity of bacterial respiration.

### 2.6. The Effect of NC on the Growth and Development of Glycine max L.

**Figure 13:**
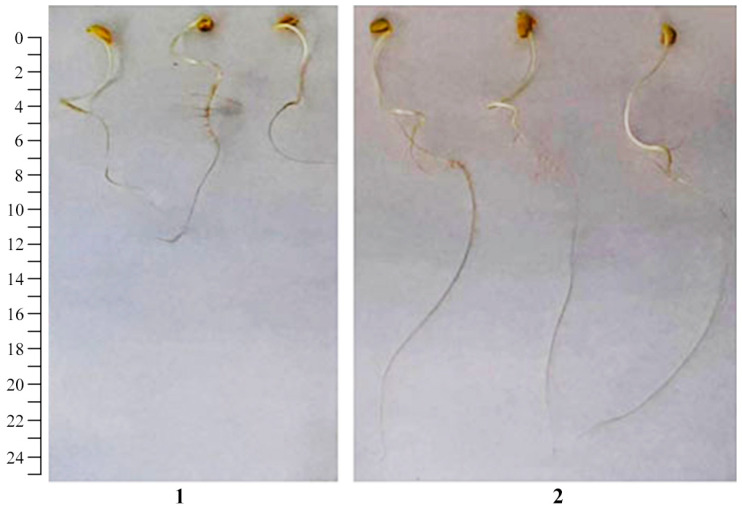
Germination of soybean seeds (**1**) control group and (**2**) under the influence of AGr-Cu. The scale size is 25 cm.

A study was carried out on the resulting AGr-Cu as a potential growth stimulator in vitro when treating soybean seeds *Glycine max* L. At the end of the experiment after 7 days of germination of soybean seeds, the results showed the effect of the NC on morphometric parameters (Figure 13, Table 4). Thus, the AGr-Cu composite had an effect on increasing the length of the stems, as well as the mass of the roots and stems compared to those of the control. However, the NC had no stimulating effect on root length (Table 4).

At the next stage, the DC content in the tissues of soybean seedlings was determined, since LPO products are a consequence of oxidative stress and ultimately lead to complete destruction of membranes and cell apoptosis. The results of the experiment showed that in the case of AGr-Cu in the stem tissues, there was a slight decrease in DC content compared to that in the control (Table 4). In root tissues, AGr-Cu did not cause an increase in DC content.

## 3. Discussion

The supramolecular organization of the resulting NC is represented as a set of nanocrystallites evenly distributed in a polysaccharide matrix, in which the nanoparticles are sterically and coordinately stabilized. The discussed AGr itself represents a complex intertwined helical macromolecular system [57]. And nanocrystallites in NC in TEM micrographs are predominantly spherical particles (electron-dense dark spots), which are spatially separated by the matrix at distances equal to or greater than their diameter and are uniformly dispersed in AGr (Figure 2). The nanocrystallite size distribution in AGr-Cu varies narrowly over a range with a predominant average size of 3 ± 1 nm, as confirmed by XRD analysis (Figure 3A).

An intense EPR signal characteristic of Cu^+2^ indicates a partial, deeper oxidation of copper (Figure 4), which is also formed in the resulting nanoparticles, primarily apparently on their surface. In recent years, studies have shown the partial spontaneous transformation of copper-containing nanoparticles during the oxidation of Cu on the surface of the particles [58]. And thus, according to the spectral characteristics, Cu^+2^ also forms characteristic coordination bonds with oxygen-containing groups (such as −OH, −CH_2_OH, etc.) of the polysaccharide matrix or flavonoids in AGr [4,38,50,59].

A series of experiments to study the presence of biological activity of the NC showed that the copper-containing composite did not have a negative effect on the growth and development of plants. Moreover, the addition of AGr-Cu to the potato growing environment promoted the development of a strong root system (Table 1). An increase in root formation under the influence of metal-containing, including copper-containing, nanocomposites is a well-known effect, which is confirmed by various literature data [4,60,61]. There are many scientific manuscripts devoted to studying the influence of various nanoparticles, including Cu_2_O, on the morphological parameters of plants. Thus, it is worth noting the results showing a positive effect of copper-containing nanoparticles on the development of various agricultural plants and stimulation of their protective functions [62,63,64,65], for example, an increase in the activity of protective proteins, the rate of organogenesis, fungicidal activity, etc. The review by Prof. Feigl [66] notes that copper oxide nanoparticles promote plant growth and development and improve photosynthesis processes, nutrient absorption, and root growth, but high concentrations of Cu can lead to the suppression of many biochemical processes. A number of studies have shown that Cu and its oxide nanoparticles can have a toxic effect on plants, the root system in particular [51,60,67].

The results showed that the roots of various plants can become brittle or their weight decreases with increasing Cu content in the nutrient medium. This is primarily due to the need to maintain the concentration of Cu in the nutrient medium and provide mineral fertilizers for the plant in safe doses. It is important to note that the results of our experiments did not show a negative effect on potato development, including metal accumulation in plant organs (Table 2). The use of AGr-Cu shows that the solution to such pressing issues is possible with the use of similar organomineral nanosystems in compliance with the concentration standards in the plant nutrient medium.

Studies on the stress resistance of potato plants have shown that both in plants infected by *Cms* and in the presence of AGr-Cu, the DC content in the tissues of roots and leaves decreased compared to that in the control (Figure 8), which may be due to the action of the immune system of resistance to stress of potato *Solanum tuberosum* L. At the same time, a decrease in ROS under the influence of the NC is also observed (Figure 7), which is probably due to the antioxidant effect of nanoparticles [4,68,69,70], it is explained by single-electron transfer, the mechanism of which is discussed in review [68]. Such inorganic nanoparticles, including nanocrystallites of copper oxides [48], can have pronounced antioxidant activity. It is also important to note that the flavonoids contained in AGr themselves act as spin traps and are capable of intercepting ROS [71], which may make an additional contribution to the effect of antioxidant activity. Regarding the study of catalase activity, the results show (Figure 9) that in plant tissues, the activity of this enzyme in the variant with AGr-Cu is higher than that in the control. A similar increase in the activity of AOS enzymes in plant tissues was previously observed under the influence of selenium nanoparticles [72]. Catalase performs catalytic functions in oxidative processes and plays a significant role in plant respiration. The main function of catalase is the decomposition of hydrogen peroxide into water and molecular oxygen. However, catalase is most active in young viable plant tissues and organs. With the aging of tissues, as well as with a decrease in their viability, the activity of this enzyme naturally decreases. Also, the importance of catalase functioning lies in the adaptation of plants to environmental conditions [73]. The potato plants used in the experiments were quite young, and when AGr-Cu is added, new environmental conditions are created to which the plant organism needs to adapt. It can be assumed that these factors primarily influence the increase in catalase content in potato tissues in vitro.

When studying the biological activity, one of the main tasks was to evaluate the antibacterial effect of the resulting NC on the viability of the phytopathogen *Cms*. A number of studies have shown the success of using Cu-containing nanoparticles as antibacterial agents, including in suppressing the dehydrogenase activity of pathogenic bacteria [74,75]. The results of the experiments on antibacterial activity showed the manifestation of the bacteriostatic ability of AGr-Cu against the bacterium *Cms* (Figure 12A). On the other hand, another important characteristic of high bacterial survival is their dehydrogenase activity [56], and the experiment revealed a pronounced effect of suppressing this activity in the presence of AGr-Cu (Figure 12B). A similar effect was found on biosynthesized Cu-containing nanoparticles with sizes from 5 to 295 nm, which showed high antimicrobial activity, suppressed the growth of pathogenic bacteria and fungi, and also increased plant germination and growth [76].

A study of the growth-stimulating effect when treating soybean seeds *Glycine max* L. showed that AGr-Cu actually has a positive effect on the development of seedlings. The NC stimulates the germination of soybean seeds (Figure 13, Table 4), promotes development, and does not have a negative effect on the status of the redox system of plants, even slightly reducing the DC content (Table 4). In comparison with our previous results on similar nanosystems, we note that AGr-Cu did not have a pronounced effect on the development and growth of the photosynthetic part of plants, for example, in comparison with manganese-containing nanocomposites [4,12]. However, the AGr-Cu promoted the development of the root system and also had a pronounced effect on the germination of soybean seeds *Glycine max* L. With regard to the antibacterial activity, there was also significant suppression of the phytopathogen *Cms*, and the effect is comparable to selenium-, silver-, and manganese-containing nanocomposites [4,32,33,77]; moreover, AGr-Cu greatly reduced the dehydrogenase activity of *Cms*. Thus, the experiments conducted indicate that the use of Cu-containing NCs based on the natural polysaccharide AGrarepromising as a new potential multifunctional agent for agricultural plants, as well as in biotechnology for artificial nutrient media in the development of autonomous life support systems.

## 4. Materials and Methods

### 4.1. Synthesis of Copper-Containing Nanocomposites

The AG was obtained from a polysaccharide of Siberian larch, *Larixsibirica* Ledeb. (Wood Chemistry Ltd., Irkutsk, Russia) [17].Earlier, we studied the effect of original polysaccharide on potatoes (*Solanum tuberosum* L.) and bacteria, where the used polysaccharide did not have a negative effect on plants, and even stimulated the growth of the *Cms* colonies and rhizosphere bacteria [32,77].

AGr-Cu was synthesized according to the following procedure: 2 mL of an aqueous solution containing 0.09 g CuCl_2_ × 2H_2_O was added to a solution of 1 g of AGr (18 kDa) in 6 mL of water with vigorous stirring, kept for 30 min at 47 °C, then 5 mL of an aqueous solution was added containing 0.08 g NaBH_4_ and 0.003 g NaOH. The reaction mixture was kept for 3 h with vigorous stirring and filtered through a paper filter. The target product was isolated from the filtrate and purified from low-molecular impurities by double reprecipitation from ethanol and dried in vacuum.

The obtained NCs were fine brown-colored powders with the color intensity being dependent on the mass content of Cu in the sample. The average mass value of Cu in nanobiocomposite AGr-Cu was 7.5 wt%.

### 4.2. Physicochemical Measurements

To study the morphology of AGr-Cu films, the obtained NC was dissolved in water. Then, its water solution was applied to grids with formvar supports and dried. The prepared samples of NC films were examined using a Leo 906 E (Carl Zeiss, Jena, Germany) TEM at an accelerating voltage of 80 kV. Micrographs were taken with a MegaView II camera (Münster, Germany) and processed using Mega Vision software v. 1.34.

Elemental analysis of the obtained NC was performed on a Flash EA 1112 CHNS-O/MAS 200 analyzer (Waltham, MA, USA); also, the percentage of Cu in the AGr-Cu was determined using the energy-dispersive X-ray spectroscopy function built into a scanning electron microscope (SEM) TM 3000 (Hitachi, Tokyo, Japan) chamber equipped with SDD XFlash4304-H detector (Tokyo, Japan) for imaging, where the sample was subjected to electron impact. Atoms of the sample were excited via electron beam and, thus, emitted X-rays of wavelengths characteristic of each chemical element. Analyzing the energy spectrum of X-ray emissions, we assessed the sample’s qualitative and quantitative composition. The number of repetitions for each sample was five, as well as five measurement areas in each sample.

Molecular weight was determined using gel permeation chromatography on a column filled with Sephadex G-100 (24 × 350 mm). Dextran (20, 40, 100, and 2000 kDa) and D-galactose standards were used for calibration. An isotonic solution was used as an eluent.

The FTIR spectra were recorded on a Bruker Vertex 70 FT-IR instrument (Billerica, MA, USA) in KBr pellets.

XRD analysis was performed on the “Bruker” D8 ADVANCE (Billerica, MA, USA) powder diffractometer (Cu radiation). The estimated sizes of metal nanocrystallites were determined by XRD analysis, and their linear dimensions were calculated by the Scherrer formula [43].

The percentage of Cu in the plant material was determined by atomic absorption analysis using a Hitachi FlexSEM 1000 II (Hitachi, Tokyo, Japan) SEM equipped with an EDX AzTec energy-dispersive accessory for elemental analysis. Elemental analyses were performed on the tissues of roots, stems, and leaves for each series of plants grown on a nutrient medium with AGr-Cu to compare with the control, according to the literature method [78]. The biological material was dried in an SSh-80-02 SPU (Smolensk, Russia) drying cabinet at 60 °C for 32 h and then calcined in a LOIP LF-5/11-G1 muffle furnace (Moscow, Russia)at 600 °C for 3 h. The obtained ash was analyzed for metal content. The number of repetitions for each sample was five; the areas of measurement, five for each sample.

The EPR spectra were recorded on a FT Bruker ELEXSYS E-560 spectrometer, Billerica, MA, USA (X-band 9.7 GHz). CW EPR spectra were recorded under the following conditions (in quartz ampoules with a diameter of 3 mm): modulation amplitude, 1–10 Gs; frequency modulation, 100 kHz; time constant, 0.02 s; conversion time, 0.06 s; microwave power, 0.6325 mW; average number of scans, 20; amplification, 50 dB at room temperature.

### 4.3. Plant Material

Plants of the Lukyanovskii potato variety, which are susceptible to biotic stresses, were used in vitro in the study [79]. Potato plants were cultivated under factor-static conditions on the Murashige–Skoog growth medium (Sigma-Aldrich, Inc., St. Louis, MO, USA), where Cu was added in the form of Cu sulfate CuSO_4_ × 5H_2_O.

To study the effect of natural polysaccharide-based NC on the growth and productivity of potato, the plants were cultivated under factor-static conditions on the Murashige–Skoog liquid nutrient medium, CuSO_4_ × 5H_2_O being replaced by AGr-Cu. The amount of the composite was determined according to the required content of Cu in the medium according to the prescription based on the mass fraction of the metal in NC (based on the mass amount of elemental Cu).

The cuttings of potato were planted to the internode depth in agar nutrient medium and cultivated for 42 days under factor-static conditions at 24–25 °C, illumination of 5–6 Klx, and a photoperiod of 16 h, periodically measuring the length and counting the number of leaves. At the end of the experiment, the biomass of the above-ground part and the biomass of the roots were determined, and physical–chemical properties of the plant material were analyzed. Independent experiments were repeated three times; in each experiment, ten plants were grown. For physical–chemical study, three plants from each experiment were used.

The content of photosynthetic pigments was determined spectroscopically. Pigments were extracted with 80% acetone, and their quantity was determined using a Specord S 100 spectrophotometer (Analytik Jena, Jena, Germany). The content of chlorophyll and carotenoids was calculated using the formulas of Vernon and Wettstein per unit leaf fresh weight [80]. A sample of fresh leaf tissue was homogenized with 10 mL of a mixture of petroleum ether and acetone, followed by additional extraction of pigments with undiluted acetone. After combining the extracts, the mixture was brought to 25 mL by adding acetone, and the optical density of the resulting solution was measured at 662 and 644 nm.

Determination of the viability of potato root tissues in vitro was carried out using a double-staining method with fluorescent dyes: FDA at a final concentration of 50 µM (Sigma-Aldrich, Inc., St. Louis, MO, USA) and PI at a final concentration of 7.5 µM (Biotium, Fremont, CA, USA). Potato plants in vitro were transferred to Murashige–Skoog liquid nutrient medium. After 3 days, aqueous solutions of AGr-Cu and a suspension of *Cms* were added to the plant growth medium. Plants were incubated with AGr-Cu and *Cms* for 3 days, then the viability of root tissues was determined. To do this, a potato root was placed in 200 μL of liquid Murashige–Skoog medium and incubated with the above dyes for 5 min at 26 °C. FDA is a vital dye and is initially a non-fluorescent compound. FDA penetrates the cell membranes of a living cell, where, due to the activity of endogenous esterases that cleave the diacetate residue, it is converted into fluorescein, after which it acquires the ability to fluoresce in the green channel, which was assessed using Filter set 10 (EX BP 450–490, BS FT 510, EM BP 565). Dead cells lack esterase activity, so no staining occurs. PI penetrates into dead and dying cells in which the permeability of the plasma membrane is impaired, it combines with DNA, and it forms 52 bonds between bases (one dye molecule per 4–5 base pairs). At the same time, the ability of PI to fluoresce in the red channel using Filter set 15 (EX BP 546/12, BS FT 580, EM LP 590) increases several times. The dye can also interact with RNA molecules. To quantify live (FDA green-stained) and dead (PI red-stained or unstained) cells, 10 random fields of view obtained using a fluorescence microscope were assessed in each experiment [81].

The studies were carried out on soybean seedlings *Glycine max* L. (the Sayana variety) with increased cold and frost resistance. The Sayana variety belongs to the early ripeness group and ripens in 98 days. The height of this plant variety is up to 105–147 cm. The developed soybean variety of the northern ecotype Sayana is characterized by significant resistance to low air temperatures and increased productivity in long-day conditions, insufficient heat supply, and cold stress [82,83]. The experiments were carried out in three biological and three analytical replicates.

To study the effect of AGr-Cu on the intensity of seed germination, a series of experiments were carried out in which 10 soybean seeds were used on a substrate. For disinfection, the seeds were placed in a glass with 96% ethanol for 1 min, and then the seeds were placed in a glass with 3% hydrogen peroxide for 20 min, followed by washing the seeds 3 times in water. An aqueous solution of NC was prepared in 30 mL of water with 300 μL of AGr-Cu solution, while the seeds were soaked in the AGr-Cu solution for 30 min and then dried; the control was soaked in water for 30 min. Then, the seeds were transferred to a Petri dish (with a disk of moistened filter paper) in three variants for AGr-Cu and control. The 2 filters were placed in prepared Petri dishes, 5 mL of sterile water was poured to wet them, then the dishes were placed in a thermostat for seed germination at a temperature of 23 °C. After 2 days, 5 mL of sterile water was added to each Petri dish to moisten the seeds, and the incubation continued in the thermostat. After 7 days of seed germination, biometric and biochemical parameters (DC content in seedling tissues) were measured.

### 4.4. Stress Resistance Experiments

The content of ROS in potato roots was determined spectrophotometrically using a xylenol orange dye [52]. The determination of the primary products of DC in the tissues of potato plants was carried out according to the standard method using hexane and isopropanol in 30 and 60 min after the addition of AGr-Cu solution into the potato growth medium in vitro [84,85]. The peroxidase activity in potato tissues was determined according to the Boyark in method [86]. The analysis of catalase activity in plants was carried out using spectrophotometry by carrying out a color reaction between hydrogen peroxide and ammonium molybdate with the measurement of the optical density of the reaction products at λ = 470 nm [87]. The primary products of LPO and DCs in shoot and root tissues of soybean seedlings were determined using hexane and isopropanol [88]. The concentration of MDA was determined by the method using 20% trichloroacetic acid and 0.5% thiobarbituric acid solution [89].

### 4.5. Bactericidal and Bacteriostatic Effect of the AGr-Cu

The strain Ac-1405 of Gram-positive bacterium *Cms* causing potato ring rot was used in this study. It was obtained from the All-Russian collection of microorganisms (GK Skryabin Institute of Biochemistry and Physiology of Microorganisms, Russian Academy of Sciences, Pushchino, Moscow Region), and the strain was cultivated on GPY medium [90] containing yeast extract 5 g/L (Sigma-Aldrich, Inc., St. Louis, MO, USA), glucose 5 g/L (Diaem, Moscow, Russia), peptone 10 g/L (Sigma-Aldrich, Inc., St. Louis, MO, USA), NaCl 5 g/L (NevaReaktiv, Saint Petersburg, Russia), and agar 7 g/L (Diaem, Moscow, Russia). To maintain the culture, the tubes with slant agar were placed in a thermostat at 25 °C. For the experiment, the bacterial colony was transferred from a solid nutrient medium to a liquid one and grown for 2 days.

A 0.05% aqueous solution of AGr-Cu and their precursors were added to flasks with a bacterial suspension (optical density D = 0.9). The solutions of all compounds were preliminarily subjected to cold sterilization (syringe nozzle “Minisart NML”, pore size 0.22 µm). The concentration of the active substance was equal for all compounds: 0.000625% and 0.00625% (based on the mass amount of Cu in the precursor). When choosing the effective concentrations, we were guided by the concentrations used for selenium- and manganese-containing NCs based on polysaccharides;in previous experiments, AGr-Cu was used at a final concentration of 0.000625% selenium/manganese in a solution [4,12,77,91]. To study the bacteriostatic activity of AGr-Cu against bacteria, a liquid culture of microorganisms was grown in the dark at 26 °C under aerated conditions (80 rpm) in flasks containing a GPY nutrient medium (pH 7.2). After addition of the composite, the optical density of the suspension was measured at 595 nm on a Bio-Rad spectrophotometer model 680 (Bio-Rad Laboratories, Inc., Hercules, CA, USA) after 24 h of co-incubation.

To determine the dehydrogenase activity of *Cms* cells, 2,3,5-triphenyltetrazolium chloride (TTC) was used. TTC is able to bind with reducing agents formed during the respiration of NAD(P)H_2_, forming an insoluble precipitate of intense crimson color. The bacterial suspension was incubated with AGr-Cu for 24 h. Then, the bacterial suspension was centrifuged in an Eppendorf microcentrifuge for 5 min at 16,000 rpm; after draining the liquid, the remaining sediment was filled with a 0.5% TTC solution and left for 3 h in the dark at 26 °C. The liquid was removed, and the precipitate was washed with water 2 times, filled with ethanol, and placed in a bath for 15 min at 60 °C. The optical density of the formazan solution was measured at 490 nm on Bio-Rad spectrophotometer model 680 (Bio-Rad Laboratories, Inc., Hercules, CA, USA) [92].

Statistical data processing was carried out using the SigmaPlot v.12.5 program (SYSTAT Software, Chicago, IL, USA). The data obtained after treatment were statistically compared with controls using the nonparametric Mann–Whitney U test, Kruskal–Wallis test, and Fisher’s exact test.

## 5. Conclusions

As a result, we have obtained a Cu-containing NC based on the natural polysaccharide AGr as a universal trophic low-dose microfertilizer, a safe and biodegradable carrier of mineral microelements with biologically active properties for plant protection. AGr-Cu had no toxic effect on potatoes, and plants grown on a medium with the NC showed results comparable to those of the control in elemental analysis, as well as better results in the biometric parameters of the root system. Growing plants in a medium with AGr-Cu improved the protective functions of the plants compared to those of the control. Also, among the most important results, it is worth noting that the resulting AGr-Cu has an antibacterial effect against the phytopathogenic bacterium *Cms*. The observed effect is probably explained by the synergistic effect of the bioactivity of the polysaccharide used, enriched with flavonoids, and copper oxide nanoparticles. In addition, AGr-Cu had a positive growth-stimulating effect when treating soybean seeds, without causing a negative effect on the status of the plant redox system. Thus, the results showed the promise of using such Cu-containing nanosystems as novel multifunctional agents in agriculture and biotechnology for artificial nutrient media, including in the development of autonomous life support systems. In addition, further research will involve testing the resulting nanosystem under the field conditions to create a promising new generation multifunctional fertilizer for agriculture.

## Figures and Tables

**Table 1:** Mass of the roots and vegetation part in the control and in plants grown in a nutrient medium with AGr-Cu.

Treatment	Roots	Vegetation Part
Control	0.144 ± 0.022	0.346 ± 0.019
AGr-Cu	0.154 ± 0.022	0.344 ± 0.029

**Table 2:** Accumulation of Cu(wt% ± SD) in potato organs grown in a medium with AGr-Cu in comparison with that in the control.

Treatment	Roots	Stems	Leaves
Control	0.15 ± 0.06	0.07 ± 0.06	0.02 ± 0.01
AGr-Cu	0.09 ± 0.04	0.03 ± 0.01	0.05 ± 0.03

**Table 3:** Effect of AGr-Cu on pigment concentration (mg/L) in leaf tissues of potato plants in vitro.

Treatment	Chlorophyll *a*	Chlorophyll *b*	Chlorophyll *a* and *b*	Caratenoid
Control	0.80 ± 0.03	0.55 ± 0.08	1.35 ± 0.33	1.30 ± 0.24
*Cms*	0.53 ± 0.01 *	0.17 ± 0.06 *	0.70 ± 0.16 *	1.22 ± 0.17
AGr-Cu	1.00 ± 0.02 *	0.48 ± 0.01	1.44 ± 0.19	1.31 ± 0.03
AGr-Cu + *Cms*	0.72 ± 0.03	0.37 ± 0.06	1.08 ± 0.06	1.41 ± 0.06 *

The (*) symbol marks significant differences according to the Mann–Whitney test (*p* ≤ 0.01).

**Table 4:** Effect of AGr-Cu on morphometric parameters and DC content in the tissues of soybean seedlings compared to control.

Treatment	Length, cm		Mass, g		Content of DC, nmoles/g Wet Mass	
	Root	Stem	Root	Stem	Root	Stem
Control	11.43 ± 1.11	4.59 ± 0.28	0.065 ± 0.007	0.085 ± 0.005	5.45 ± 0.73	6.06 ± 1.01
AGr-Cu	11.12 ± 1.12	5.78 ± 0.35 *	0.124 ± 0.007 *	0.122 ± 0.011 *	1.52 ± 0.13 *	3.68 ± 1.07

The (*) symbol marks significant differences according to the Mann–Whitney test (*p* ≤ 0.01).

## Data Availability

Data are contained within the article.
